# Neurite orientation dispersion and density imaging reveals white matter microstructural alterations in adults with autism

**DOI:** 10.1186/s13229-021-00456-4

**Published:** 2021-06-30

**Authors:** Christina Andica, Koji Kamagata, Eiji Kirino, Wataru Uchida, Ryusuke Irie, Syo Murata, Shigeki Aoki

**Affiliations:** 1 Department of Radiology, Juntendo University Graduate School of Medicine, Tokyo, Japan; 2 Department of Psychiatry, Juntendo University Graduate School of Medicine, Tokyo, Japan; 3 Department of Psychiatry, Juntendo University Shizuoka Hospital, Shizuoka, Japan; 4 Department of Radiological Sciences, Faculty of Healthy Sciences, Komazawa University, Tokyo, Japan

**Keywords:** Autism, Diffusion tensor imaging, Linear discriminant analysis, Neuronal loss, Neuroinflammation, Neurite orientation dispersion and density imaging, Region-of-interest, Tract-based spatial statistics, White matter microstructure

## Abstract

**Background:**

Evidences suggesting the association between behavioral anomalies in autism and white matter (WM) microstructural alterations are increasing. Diffusion tensor imaging (DTI) is widely used to infer tissue microstructure. However, due to its lack of specificity, the underlying pathology of reported differences in DTI measures in autism remains poorly understood. Herein, we applied neurite orientation dispersion and density imaging (NODDI) to quantify and define more specific causes of WM microstructural changes associated with autism in adults.

**Methods:**

NODDI (neurite density index [NDI], orientation dispersion index, and isotropic volume fraction [ISOVF]) and DTI (fractional anisotropy [FA], mean diffusivity [MD], axial diffusivity, and radial diffusivity [RD]) measures were compared between autism (*N* = 26; 19 males and 7 females; 32.93 ± 9.24 years old) and age- and sex-matched typically developing (TD; *N* = 25; 17 males and 8 females; 34.43 ± 9.02 years old) groups using tract-based spatial statistics and region-of-interest analyses. Linear discriminant analysis using leave-one-out cross-validation (LDA-LOOCV) was also performed to assess the discriminative power of diffusion measures in autism and TD.

**Results:**

Significantly lower NDI and higher ISOVF, suggestive of decreased neurite density and increased extracellular free-water, respectively, were demonstrated in the autism group compared with the TD group, mainly in commissural and long-range association tracts, but with distinct predominant sides. Consistent with previous reports, the autism group showed lower FA and higher MD and RD when compared with TD group. Notably, LDA-LOOCV suggests that NDI and ISOVF have relatively higher accuracy (82%) and specificity (NDI, 84%; ISOVF, 88%) compared with that of FA, MD, and RD (accuracy, 67–73%; specificity, 68–80%).

**Limitations:**

The absence of histopathological confirmation limit the interpretation of our findings.

**Conclusions:**

Our results suggest that NODDI measures might be useful as imaging biomarkers to diagnose autism in adults and assess its behavioral characteristics. Furthermore, NODDI allows interpretation of previous findings on changes in WM diffusion tensor metrics in individuals with autism.

## Background

Autism is a neurodevelopmental condition characterized by social communication and interaction deficits and the presence of restricted and repetitive patterns of behaviors, interests, or activities [1]. Evidences suggesting the association between behavioral anomalies in autism and white matter (WM) microstructural alterations that persist until adult life are increasing [2–6]. Although the pathophysiological causes of autism are not yet fully understood, recent postmortem studies support the view that neuronal loss [7, 8] and neuroinflammation [9, 10] contribute to autism etiology.

Diffusion tensor imaging (DTI) has shown great potential in noninvasively probing WM tissue microstructure by enabling the measurement of the diffusion properties of water in the tissue. DTI-derived measures such as fractional anisotropy (FA), mean diffusivity (MD), axial diffusivity (AD), and radial diffusivity (RD) characterize the degree of anisotropy of water molecules, overall magnitude of diffusion, diffusional directionality perpendicular to the axon, and diffusional directionality along the axon, respectively [11]. Decreased FA accompanied by increased MD and RD has been reported in the WM tracts involved in social processing in individuals with autism [12, 13]. Alterations are demonstrated in the corpus callosum (CC), arcuate fasciculus, inferior longitudinal fasciculus (ILF), inferior fronto-occipital fasciculus (IFOF), superior longitudinal fasciculus (SLF), and uncinate fasciculus (UF) [12]. The role of these WM tracts in autism-related behaviors is summarized in Table 1. However, due to the lack of specificity, the underlying pathology of reported differences in DTI measures in autism remains poorly understood. Lower FA and higher MD indicate impaired WM integrity owing to changes in the axonal diameter, fiber density, tissue geometry, myelination degree, and an increase in extracellular free-water [14, 15]. Higher AD and RD appear to be modulated by axonal loss and demyelination, respectively [16]. However, the interpretations may be meaningless because pathology might change the diffusional directionality according to the underlying structures [17].

**Table 1:** The anatomical definition and functions of white matter tracts frequently involved in autism

White matter tracts	Category	Connection	Function related to autism
ATR	Projection fiber	Dorsomedial thalamic nucleus to prefrontal cortex through the anterior limb of internal capsule	Executive function and planning complex behaviors
Corpus callosum	Commissural fiber	The cortices of the two cerebral hemispheres	Cognitive and social function
Cingulum	Long-range associative fiber	Cingulate gyrus to the anterior thalamic nuclei	Visuospatial processing and memory access
IFOF	Long-range associative fiber	Occipital cortex, temporo-basal areas, and superior parietal lobe to the frontal lobe	Semantic processing
ILF	Long-range associative fiber	Occipital lobe to the anterior temporal lobe	Face and emotion recognition
SLF	Long-range associative fiber	Frontal lobe to parietal lobe and partially to temporal lobe	Visuospatial attention, language auditory comprehension, articulatory processing, reading, and lexical access
UF	Long-range associative fiber	Lateral orbitofrontal cortex and Broadmann area 10 to anterior temporal lobes	Episodic memory, language, and social emotional processing

ATR, anterior thalamic radiation; IFOF, inferior fronto- occipital fasciculus; ILF, inferior longitudinal fasciculus; SLF, superior longitudinal fasciculus; UF, uncinate fasciculus

Novel advanced diffusion-weighted imaging techniques aim to improve WM characterization by employing multi-compartment models to describe various WM features. One of these approaches is the neurite orientation dispersion and density imaging (NODDI), which incorporates multiple shells with different b-values to model the brain tissue into three compartments showing different diffusion properties with a clinically feasible protocol (imaging the whole brain within 30 min in the original protocol [18] and 13 min in the present study). In the NODDI model, each voxel is assumed as a combination of three compartments: intracellular (modeled as restricted anisotropic non-Gaussian diffusion), extracellular (modeled as hindered anisotropic Gaussian diffusion), and cerebrospinal fluid (CSF; modeled as isotropic Gaussian diffusion). NODDI can disentangle the different microstructural contributions to DTI measures, consequently, providing more specific insights into the underlying WM microstructural changes. NODDI-derived measures, including neurite density index (NDI), orientation dispersion index (ODI), and isotropic volume fraction (ISOVF), reflect neurite density, neurite orientation dispersion, and extracellular free-water, respectively [18]. In brief, lower values of NDI represent lower neurite density (or packing of neuronal tissue), whereas higher ODI indicates fanning of neurites, and ISOVF measures the extracellular component of the free-water compartment [18]. Previous studies using multishell diffusion-weighted imaging techniques (including NODDI) have detected reduced neurite density in the gray matter (GM) of children with autism and brain areas related to facial emotion recognition in young adults with autism [19, 20].

In the current study, we employed NODDI (1) to assess differences in the WM microstructure integrity in adults with autism compared with typically developing (TD) individuals; (2) to define more specific causes of WM microstructural alterations in autism by hypothesizing that NODDI might resolve component pathologies of autism in the WM, such as neuronal loss or neuroinflammation, in order to overcome DTI limitations; and (3) to determine if these WM properties are associated with autism-related scores. Most previous studies in adults with autism have focused on evaluating the changes in the microstructure of WM [6, 21–24] or the morphology of GM [25–27]; however, the relationship between the different brain regions and autism remain unclear. In addition, GM structural analysis was also performed to observe the association between WM microstructural alterations measured using NODDI and GM structural changes. Finally, a linear discriminant analysis (LDA) [28] using leave-one-out cross-validation (LOOCV) was implemented to delineate individuals with autism and TD and understand the discriminative power of diffusion measures.

## Methods

### Study participants

The study protocol was approved by the research ethics committee of Juntendo University Hospital in Tokyo, Japan, and written informed consent was obtained from each participant. A total of 51 right-handed young (18–35 years) and middle-aged (36–55 years) adults were included in this study [29]. Study participants were divided into autism (*N* = 26, 19 males; mean age 32.93 ± 9.24 years, range 19.29 − 52.93 years) and TD (*N* = 25, 17 males and 8 females; mean age 34.43 ± 9.02 years, range 20.45 − 49.21 years) groups. Individuals with autism were recruited from the outpatient clinics of Juntendo Koshigaya Hospital (Saitama, Japan) and Juntendo Shizuoka Hospital (Shizuoka, Japan), and TD individuals were recruited from the same hospitals’ staff.

Autism diagnosis was based on the fifth edition of the Diagnostic and Statistical Manual of Mental Disorders [1]. Each participant was assessed using the autism-spectrum quotient (AQ) [30], empathizing quotient (EQ) [31], and systemizing quotient (SQ) [32]. AQ, EQ, and SQ are self-administered measures (for use with adults of normal intelligence) of the autistic traits, social functioning, and capability to analyze or construct systems, respectively. AQ comprises five subscales: social skills, attention switching, attention to detail, communication skills, and imagination. TD participants had no history of any psychiatric, neurological, or developmental disorders. None of the participants reported a history of head injury. A summary of demographic and autism-related scores is presented in Table 2.

**Table 2:** Demographic characteristics of study participants

	Autism (*N* = 26)	TD (*N* = 25)	*P*-value
Age (mean ± SD, range; years)	32.93 ± 9.24, 19.29−52.93	34.43 ± 9.02, 20.45−49.21	0.56
Sex (male/female)	19/7	17/8	0.69
Years of education (mean ± SD, range)	14.67 ± 2.28, 12−19	15.32 ± 2.36, 12−19	0.33
Clinical scores:			
AQ (mean ± SD, range)			
Total	32.92 ± 5.19, 22−41	15.16 ± 5.41, 5−26	< 0.0001
Social skill	6.96 ± 1.61, 3−9	2.40 ± 2.02, 0−8	< 0.0001
Attention switching	7.27 ± 1.82, 3−10	3.28 ± 1.90, 0−7	< 0.0001
Attention to detail	4.96 ± 1.93, 2−9	4.28 ± 2.13, 0−8	0.24
Communication	7.31 ± 1.83, 3−10	2.00 ± 1.80, 0−6	< 0.0001
Imagination	6.42 ± 1.60, 4−9	3.20 ± 1.58, 1−7	< 0.0001
EQ (mean ± SD, range)	24.50 ± 7.42, 11−35	38.56 ± 10.76, 23−60	< 0.0001
SQ (mean ± SD, range)	26.73 ± 14.32, 5−59	22.40 ± 9.76, 1−47	0.22
Global brain volumes			
ICV (mean ± SD; mL)	1415.94 ± 218.66	1318.14 ± 258.43	0.15
Normalized WM volume (WM volume /ICV)	0.34 ± 0.04	0.36 ± 0.05	0.09
Normalized GM volume (GM volume/ICV)	0.44 ± 0.06	0.48 ± 0.06	0.08

AQ, autism-spectrum quotient; EQ, empathy quotient; GM, gray matter; SD, standard deviation; SQ, systemizing quotient; TD, typically developing subjects; WM, white matter

### MRI data acquisition

MRI data of all study participants included diffusion-weighted images and three-dimensional (3D) T1-weighted images, acquired using the same 3 T Achieva scanner (Philips Healthcare, Best, The Netherlands). Multishell diffusion-weighted imaging was performed using a spin echo echo-planar imaging sequence (*b* = 1000 and 2000s/mm^2^, 32 diffusion-weighted directions, 1 *b*0 image, echo time [TE] = 100 ms, repetition time [TR] = 9810 ms, flip angle 90°, matrix size = 128 × 128, field of view [FOV] = 256 × 256, slice thickness = 2 mm), and acquisition time = 13 min, whereas 3D T1-weighted imaging was obtained using a turbo field echo (TFE) sequence with the following parameters: TE = 3.4 ms, TR = 15 ms, inversion time = 932 ms, flip angle 10°, matrix size = 256 × 256, FOV = 256 × 256, slice thickness = 1 mm, TFE factor = 116, shot interval time = 2500 ms, and acquisition time = 3.5 min.

### Diffusion-weighted image processing

All diffusion-weighted images were assessed for severe artifacts in the axial, sagittal, and coronal views. The EDDY tool, part of the FMRIB Software Library 5.0.9 (FSL, Oxford Centre for Functional MRI of the Brain, UK; www.fmrib.ox.ac.uk/fsl) [33] was used to correct eddy current-induced distortions and subject movements from diffusion-weighted data [34]. Resulting images were then fitted to the NODDI model [18] using the NODDI MATLAB Toolbox 5 (http://www.nitrc.org/projects/noddi_toolbox), and maps of NDI, ODI, and ISOVF were generated using the Accelerated Microstructure Imaging via Convex Optimization [35]. The DTIFIT tool, part of the FSL [33], was used to generate tensor-derived (FA, MD, RD, and AD) maps based on the ordinary least squares method [36] using diffusion-weighted data with *b*-values of 0 and 1000 s/mm^2^.

### Tract-based spatial statistical analysis

Voxel-wise statistical analysis of the diffusion data was carried out using tract-based spatial statistics (TBSS) [37] implemented in FSL [33]. The TBSS approach was performed to investigate DTI and NODDI measure changes between groups (autism vs. TD) and evaluate the correlation between diffusion metrics and autism-related scores in individuals with autism.

The TBSS procedure was as follows: (1) FA maps of all participants were aligned into 1 × 1 × 1 × mm^3^ Montreal Neurological Institute 152 common space (an averaged brain) using FMRIB’s nonlinear registration tool. Subsequent processing and analysis were carried out in this space for convenient interpretation and display. Notably, the following steps, i.e., creating the FA skeleton and projecting FA or other diffusion images onto the skeleton, work well at this higher resolution and limit the partial volume [37]. (2) A population-based mean FA image was created and thinned to make a mean FA skeleton, which represents centers of all tracts common to the group. This skeleton had a threshold FA level of 0.2 to exclude voxels from the GM and CSF (3). The averaged FA map of each participant was projected onto the skeleton. Other DTI (MD, AD, and RD) and NODDI (NDI, ODI, ISOVF) maps were then projected onto the FA-derived skeleton after applying each participant’s warping registration to the common space.

In autism, the pattern abnormality of WM varies across the sex of participants [38]. For exploratory purposes, TBSS analyses were also performed between male (*N* = 19, mean age 34.02 ± 7.39 years) and female (*N* = 7, mean age 29.96 ± 13.34 years) individuals with autism.

### Region-of-interest analysis

Using a region-of-interest (ROI) analysis, DTI and NODDI metrics were extracted in the genu, body, and splenium of the CC, forceps major and minor, left- and right-anterior thalamic radiation (ATR), anterior limb of internal capsule (ALIC), cingulum cingulate gyrus (CCG), corticospinal tract (CST), ILF, IFOF, SLF, and UF. The WM tracts included in this study have all been found to have significant changes in the DTI metrics in previous studies in adults with autism [21–24, 39]. Hypothesis-driven ROIs were selected to reduce the severity of correction for multiple tests that could lead to type II error (false negative) [40]. Quantitative diffusion measures (average value over the entire tract is reported) of each WM tract were obtained by first labeling the WM skeleton tract regions using the John Hopkins University’s ICBM-DTI-81 WM tractography and label atlases [41, 42].

### Brain volumes and cortical thickness measurements

Global brain [intracranial (ICV), WM, and GM] volumes and cortical thickness were measured using FreeSurfer version 6.0.0 (http://surfer.nmr.mgh.harvard.edu/fswiki). FreeSurfer was run with the “recon-all pipeline using default analysis” setting on each 3D T1-weighted image, as previously described [43].

### Statistical analysis

Independent-sample t-tests were used to compare age; years of education; AQ (total score and subscales), EQ, and SQ scores; ICV; normalized WM volume (WM volume/ICV); and normalized GM volume (GM volume/ICV). The chi-square test was used to compare the individuals according to their sex between autism and TD groups. A *P-*value of < 0.05 was considered statistically significant. These tests were conducted using SPSS Statistics for Macintosh version 25.0 (IBM, Armonk, NY, USA).

For TBSS analysis, a voxel-wise general linear model (GLM) framework, including age, sex, and ICV as covariates, was used to compare DTI (FA, MD, RD, and AD) and NODDI (NDI, ODI, and ISOVF) metrics between autism and TD groups using the FSL randomize tool with 10,000 permutations. Between-group differences were considered significant at *P* < 0.05 and corrected for multiple comparisons using the family-wise error (FWE) and threshold-free cluster enhancement approaches.

For ROI analysis, differences of diffusion measures between participants with autism and TD were performed using the GLM while controlling for age, sex, and ICV in SPSS 25. The effect size was calculated with Cohen’s *d* to evaluate the strength of the relationship in between-group comparisons [44]. The correlation analysis with autism-related scores in the autism group was performed using Pearson’s correlation coefficient. Here, the Bonferroni correction was used to correct between-group multiple comparisons (*n* = 23; forceps minor, genu, body, and splenium of the CC, forceps major, left and right ATR, CCG, CST, IFOF, ILF, SLF, and UF), with statistical significance set at *P* < 0.05/23 = 0.0022.

After comparing diffusion measures across different groups, the discriminant power of each diffusion measure was evaluated. We applied LDA using the scikit-learn package [45] in Python to identify which diffusion measure performed better in differentiating autism and TD groups. LDA is a robust classification method using a linear combination of the independent variables to predict a categorical outcome [46]. A separate LDA classifier was computed for each of the diffusion metrics, including data from the 23 ROIs described above. Since this study included a relatively small sample size, we used the leave-one-out technique to cross-validate the classification method [47].

A vertex-wise analysis on cortical volume and thickness differences between groups (autism vs. TD) was performed separately for the left and right hemispheres with FreeSurfer’s graphical user interface, query, design, estimate, and contrast using data smoothed at full-width half maximum of 10 mm. Different offsets and slope methods were used to create the design matrix while including age and sex as covariates. Multiple comparisons were corrected with a Monte Carlo simulation using a *P*-value set at < 0.05 and a cluster-wise *P-*value of 0.05 to display results.

## Results

### Study participants

Age, sex, years of education, ICV, normalized WM volume, normalized GM volume, AQ-attention to detail score, and SQ score were not significantly different between autism and TD groups (Table 2). Participants with autism had significantly (*P* < 0.0001) higher AQ (total score, social skill, attention switching, communication, and imagination domain subscales) and lower EQ scores compared to TD participants. AQ represents the degree to which a person shows autistic traits (the higher the score, the higher the degree of autistic traits) [30], whereas EQ reports the level of empathy (a lower score indicates lower empathizing skills, responsible for difficulties in social interactions in autism) [31].

### Between-group differences

**Fig. 1:**
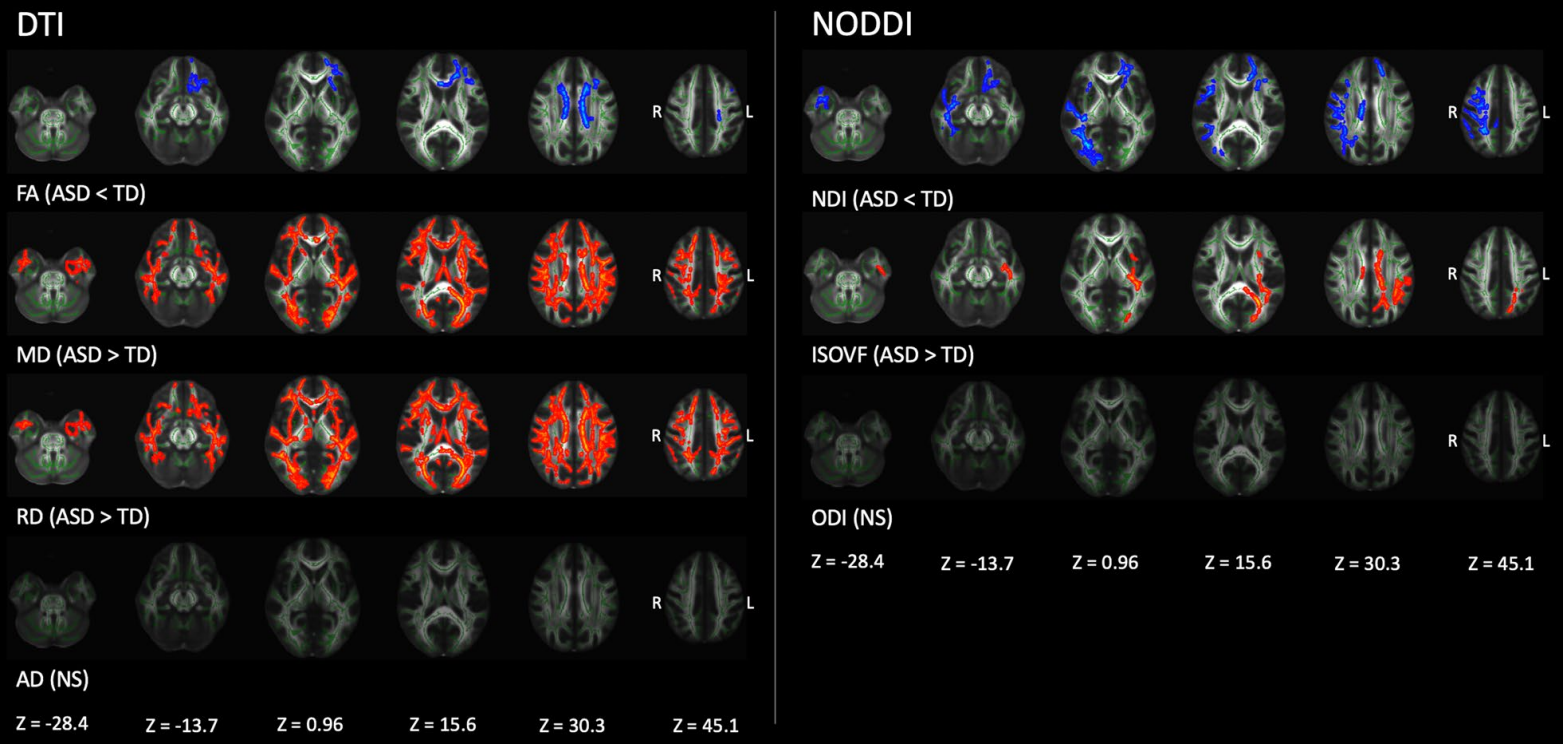
Comparison of DTI (FA, MD, AD, and RD) and NODDI (NDI, ISOVF, and ODI) measures between autism and TD groups. Using TBSS analysis, significantly (family-wise error corrected *P* < 0.05) lower FA and NDI (blue–light blue voxels) and higher MD, RD, and ISOVF (red–yellow voxels) values were observed in the autism group than that in the TD. No significant differences were observed in AD and ODI values between the groups. The FA skeleton with FA of > 0.2 is shown in green. To aid visualization, results are thickened using the fill script implemented in FMRIB Software Library. AD, axial diffusivity; DTI, diffusion tensor imaging; FA, fractional anisotropy; ISOVF, intracellular volume fraction; L, left; MD, mean diffusivity; NDI, neurite density index; NODDI, neurite orientation dispersion and imaging; NS, not siginificant; ODI, orientation dispersion index; R, right; RD, radial diffusivity; TD, typically developing

Figure 1 and Table 3 show results of TBSS analysis of the DTI and NODDI metrics. Significantly (FWE-corrected *P* < 0.05) lower FA and NDI and higher MD, RD, and ISO were demonstrated in participants with autism than in those with TD. No statistically significant differences in AD and ODI were observed between autism and TD groups. Notably, NDI changes were predominantly observed in the major WM tracts of the right hemisphere and anterior part of the left hemisphere. In contrast, ISOVF changes were demonstrated in the posterior left hemisphere. In exploratory analyses of individuals with autism, we did not find any difference in all diffusion measures between male and female groups.

**Table 3:** Comparison of tract-based spatial statistics analysis of DTI and NODDI measures in participants

Modality	Contrast	Significant voxels	Anatomical region	Mean (range) *T*-value	Peak MNI coordinates (*X*, *Y*, *Z*)
DTI					
FA	Autism < TD	4229	Bilateral ACR, SCR; Left ATR, CST, IFOF, UF, PCR; Forceps minor, genu, body, and splenium of CC	1.88 (1.07−6.09)	108, 120, 106
MD	Autism > TD	17,006	Bilateral ATR, CST, IFOF, ILF, SLF, UF, ALIC, PLIC, retrolenticular part of IC, ACR, SCR, PCR, PTR, sagittal stratum, and SFOF; Left-CCG and CgH; Right-external capsule; Forceps major and minor, genu, body, and splenium of CC, and fornix	1.61 (0.81–5.39)	48, 107, 107
RD	Autism > TD	20,728	Bilateral ATR, CST, CCG, IFOF, ILF, SLF, UF, ALIC, PLIC, retrolenticular part of IC, ACR, SCR, PCR, PTR, sagittal stratum, external capsule, and SFOF; Left-CgH; Right SLF temporal part; Forceps major and minor, genu, body, and splenium of CC, and fornix	1.61 (0.81−6.27)	144, 94, 102
NODDI					
NDI	Autism < TD	4110	Bilateral IFOF, UF, and external capsule; Left ATR, ALIC, and ACR; Right CST, ILF, SLF, retrolenticular part of IC, SLF temporal part, SCR, PCR, and PTR; Forceps major and minor, genu and body of CC	1.92 (1.07−4.84)	109, 168, 94
ISOVF	Autism > TD	2472	Left IFOF, ILF, SLF, UF, PLIC, retrolenticular part of IC, SCR, PCR, and PTR; Right-sagittal stratum; Forceps major, body, and splenium of CC	1.77 (1.04 − 4.74)	119, 101, 80

ACR, anterior corona radiata; ALIC, anterior limb of internal capsule; ATR, anterior thalamic, radiation; CCG, cingulum cingulate gyrus; CgH, cingulum hippocampus; CP, cerebral peduncle; CST, corticospinal tract; DTI, diffusion tensor imaging; FA, fractional anisotropy; IC, internal capsule; IFOF, inferior fronto-occipital fasciculus; ILF, inferior longitudinal fasciculus; ISOVF, isotropic volume fraction; MD, mean diffusivity; MNI, Montreal Neurological Institute; NDI, neurite density index; NODDI, neurite orientation dispersion and density imaging; PCR, posterior corona radiata; PLIC, posterior limb of internal capsule; PTR, posterior thalamic radiation; RD, radial diffusivity; SCR, superior corona radiata; SFOF, superior fronto-occipital fasciculus; SLF, superior longitudinal fasciculus; UF, uncinate fasciculus; TD, typically developing subjects

**Fig. 2:**
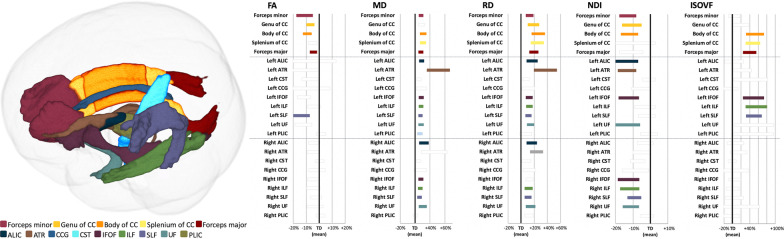
Region-of-interest analysis of WM areas involved in adults with autism. Left panel: WM areas obtained using the John Hopkins University’s ICBM-DTI-81 WM tractography and label atlases (only left side tracts are shown). Right panel: The mean of each measure in autism and TD groups (represented as the percentage difference from TD). Significant areas (Bonferroni-corrected *P* < 0.0022) are displayed in color, whereas nonsignificant tracts are shown in gray. ALIC, anterior limb of internal capsule; ATR, anterior thalamic radiation; CC, corpus callosum; CCG, cingulum cingulate gyrus; CST, corticospinal tract; FA, fractional anisotropy; IFOF, inferior fronto-occipital fasciculus; ILF, inferior longitudinal fasciculus; ISOVF, isotropic volume fraction; MD, mean diffusivity; NDI, neurite density index; RD, radial diffusivity; SLF, superior longitudinal fasciculus; UF, uncinate fasciculus; TD, typically developing

Figure 2 and Table 4 show the results of ROI analysis of the DTI (FA, MD, and RD) and NODDI (NDI and ISOVF) metrics. Significantly lower FA (*P* ≤ 0.0021, Cohen’s *d* ≥ 0.90; in the forceps minor, genu, and body of the CC, forceps major, left IFOF, and left SLF) and NDI (*P* ≤ 0.00037, Cohen’s *d* ≥ 1.01; in the forceps minor, genu and body of CC, left and right IFOF and UF, left ALIC and ATR, and right ILF and SLF) were demonstrated in autism group compared with those in TD group. Significantly higher MD (*P* ≤ 0.0015, Cohen’s *d* ≥ 1.07; in forceps minor, body and splenium of CC, forceps major, left and right ALIC, IFOF, ILF, SLF, and UF, and left ATR and the posterior limb of internal capsule), RD (*P* ≤ 0.0059, Cohen’s *d* ≥ 1.04; in forceps minor, genu, body and splenium of CC, forceps major, left and right ALIC, ATR, ILF, SLF, and UF, and left IFOF), and ISOVF (*P* ≤ 0.000032, Cohen’s *d* ≥ 1.35; in body and splenium of CC, forceps major, left IFOF, ILF, and SLF) were observed in autism group compared with those in TD group. Consistent with TBSS results, no statistically significant differences were observed in AD and ODI between autism and TD groups. LDA-LOOCV results indicated greater accuracy (82%) and specificity (NDI, 84%; ISOVF, 88%) of NDI and ISOVF compared with that of FA, MD, and RD (accuracy: 71%, 73%, and 67%, respectively; specificity: 72%, 80%, and 68%, respectively; Table 5).

**Table 4:** ROI analysis of DTI and NODDI measures in participants with autism compared to TD

	Autism		TD		*P*-value	Cohen’s d
	Mean	SD	Mean	SD		
*DTI*						
FA						
Forceps minor	0.506	0.034	0.536	0.026	0.00017	0.96
Genu of CC	0.707	0.022	0.732	0.025	0.00027	1.04
Body of CC	0.730	0.026	0.764	0.022	0.0000095	1.44
Forceps major	0.728	0.020	0.744	0.016	0.0021	0.90
Left IFOF	0.487	0.026	0.516	0.031	0.00020	1.00
Left SLF	0.479	0.034	0.521	0.033	0.000023	1.25
MD						
Forceps minor	0.776	0.024	0.745	0.021	0.000059	− 1.36
Body of CC	0.834	0.035	0.789	0.029	0.000049	− 1.39
Splenium of CC	0.782	0.030	0.742	0.026	0.000031	− 1.43
Forceps major	0.821	0.026	0.790	0.018	0.000016	− 1.38
Left ALIC	0.716	0.024	0.685	0.020	0.000024	− 1.42
Left ATR	1.038	0.138	0.889	0.140	0.00033	− 1.08
Left IFOF	0.830	0.026	0.796	0.020	0.0000089	− 1.48
Left ILF	0.862	0.027	0.828	0.021	0.000015	− 1.42
Left SLF	0.763	0.021	0.736	0.018	0.000024	− 1.40
Left UF	0.813	0.028	0.780	0.018	0.000020	− 1.39
Left PLIC	0.747	0.026	0.724	0.017	0.0015	− 1.07
Right ALIC	0.734	0.046	0.692	0.021	0.00010	− 1.19
Right IFOF	0.780	0.025	0.750	0.020	0.000027	− 1.32
Right ILF	0.789	0.024	0.762	0.020	0.000056	− 1.24
Right SLF	0.709	0.021	0.688	0.018	0.00023	− 1.08
Right UF	0.804	0.043	0.763	0.018	0.000030	− 1.24
RD						
Forceps minor	0.496	0.027	0.465	0.024	0.000068	− 1.22
Genu of CC	0.363	0.029	0.330	0.032	0.00059	− 1.07
Body of CC	0.379	0.034	0.333	0.032	0.000026	− 1.40
Splenium of CC	0.297	0.026	0.263	0.021	0.0000025	− 1.46
Forceps major	0.395	0.025	0.358	0.021	0.00000083	− 1.59
Left ALIC	0.461	0.037	0.424	0.021	0.000057	− 1.23
Left ATR	0.733	0.108	0.609	0.108	0.00011	− 1.15
Left IFOF	0.531	0.027	0.500	0.023	0.000028	− 1.26
Left ILF	0.580	0.028	0.545	0.023	0.000012	− 1.39
Left SLF	0.530	0.024	0.501	0.021	0.000085	− 1.26
Left UF	0.562	0.029	0.523	0.025	0.000012	− 1.41
Right ALIC	0.408	0.030	0.377	0.019	0.000088	− 1.25
Right ATR	0.570	0.051	0.507	0.036	0.000014	− 1.42
Right ILF	0.542	0.032	0.512	0.022	0.00027	− 1.11
Right SLF	0.474	0.024	0.449	0.023	0.00017	− 1.04
Right UF	0.505	0.034	0.470	0.022	0.000020	− 1.24
*NODDI*						
NDI						
Forceps minor	0.588	0.031	0.627	0.022	0.0000043	1.46
Genu of CC	0.651	0.039	0.687	0.032	0.00037	1.01
Body of CC	0.689	0.036	0.732	0.034	0.00022	1.22
Left ALIC	0.581	0.040	0.622	0.041	0.00023	1.01
Left ATR	0.579	0.032	0.619	0.028	0.000016	1.32
Left IFOF	0.608	0.038	0.648	0.028	0.00013	1.17
Left UF	0.587	0.045	0.629	0.032	0.00017	1.06
Right IFOF	0.567	0.037	0.604	0.028	0.00020	1.12
Right ILF	0.549	0.032	0.583	0.027	0.00035	1.12
Right SLF	0.668	0.028	0.700	0.028	0.00024	1.14
Right UF	0.489	0.024	0.518	0.031	0.00031	1.02
ISOFV						
Body of CC	0.161	0.027	0.124	0.017	0.0000018	− 1.65
Splenium of CC	0.142	0.019	0.112	0.016	0.0000011	− 1.67
Forceps major	0.137	0.017	0.113	0.014	0.00000027	− 1.56
Left IFOF	0.160	0.031	0.125	0.019	0.000032	− 1.35
Left ILF	0.134	0.026	0.102	0.019	0.000015	− 1.43
Left SLF	0.125	0.018	0.098	0.010	0.000000065	− 1.84

ALIC, anterior limb of internal capsule; ATR, anterior thalamic, radiation; CC, corpus callosum; DTI, diffusion tensor imaging; FA, fractional anisotropy; IFOF, inferior fronto-occipital fasciculus; ILF, inferior longitudinal fasciculus; ISOVF, isotropic volume fraction; MD, mean diffusivity; NDI, neurite density index; NODDI, neurite orientation dispersion and density imaging; PLIC, posterior limb of internal capsule; RD, radial diffusivity; ROI, region-of-interest; SLF, superior longitudinal fasciculus; TD, typically developing subjects; UF, uncinate fasciculus

**Table 5:** Linear discriminant analysis classification with leave-one-out cross-validation results

Modality	Accuracy (%)	Sensitivity (%)	Specificity (%)	Positive predictive value (%)	Negative predictive value (%)
DTI					
FA	71	69	72	72	69
MD	73	81	80	81	80
AD	65	58	72	68	62
RD	67	81	72	75	78
NODDI					
NDI	82	81	84	84	81
ODI	55	58	52	56	54
ISOVF	82	77	88	87	79

AD, axial diffusivity; DTI, diffusion tensor imaging; FA, fractional anisotropy; ISOVF, isotropic volume fraction; MD, mean diffusivity; NDI, neurite density index; NODDI, neurite orientation dispersion and density imaging; ODI, orientation dispersion index; RD, radial diffusivity; TD, typically developing subjects

### Correlation analysis

**Fig. 3:**
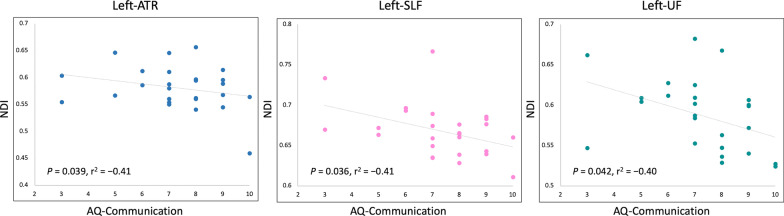
Scatterplots showing a moderate negative correlation between NDI and AQ-communication score in individuals with autism. ATR, anterior thalamic radiation; AQ, autism-spectrum quotient; NDI, neurite density index; SLF, superior longitudinal fasciculus; UF, uncinate fasciculus

NDI was moderately [48] negatively correlated with AQ-communication score in the left ATR (*P* = 0.039, *r*^2^ =  − 0.41), SLF (*P* = 0.036, *r*^2^ =  − 0.41), and UF (*P* = 0.042, *r*^2^ =  − 0.40) (Fig. 3). However, the correlation was not established after correction for multiple comparisons. No associations were found between NDI and other autism-related scores or between other diffusion measures and autism-related scores.

### Cortical volume and thickness evaluation

No statistically significant difference was observed in the cortical volume and thickness between the studied groups.

## Discussion

This study provides evidence on significant alterations in the microstructural organization of WM in young and middle-aged adults with autism. Overall, our findings confirm several previous diffusion-weighted imaging studies that have examined WM tracts in adults with autism. However, using NODDI, we were able to disentangle the contribution of the different tissue compartments underlying WM microstructural changes in autism, including neurite loss (as indexed by lower NDI) and increased extracellular free-water (as indexed by higher ISOVF), mainly in commissural and long-range association tracts that mediate autistic symptoms and traits. In addition, LDA-LOOCV results indicated that NODDI metrics (NDI and ISOVF) have higher discriminative power compared with DTI metrics (FA, MD, and RD).

Previous studies that used varied approaches to diffusion-weighted imaging data have found evidence for WM microstructural abnormalities in adults with autism in the ATR [3, 4], ALIC [23], CC [2, 4–6, 21, 22, 24, 49], cingulum [2, 3, 21, 24], forceps minor and major [21], IFOF [4, 21, 22, 49], ILF [3, 4, 21, 22, 49], SLF [3, 4, 21–23, 49], and UF [2–4, 21, 49], which is broadly neuroanatomically consistent with our findings. This shows the robustness of the diffusion MRI data analysis pipeline used in the current study. These WM tracts are known to be associated with autism behavioral characteristics. The anatomical definition of these white tracts, including the normal function and impact in autism, are summarized in Table 1 [50–56].

Our findings on reduced NDI suggest a reduction of neurite density in adults with autism [18]. Of note, NDI was well-correlated with the histological measurements of the levels of hyperphosphorylated tau protein associated with neuronal loss in a mouse model of human tauopathy (rTg4510) [57]. Consistent with our results, postmortem studies on the brain of patients with autism have previously reported reduced numbers of medium and large-caliber axons, which likely affects the synaptic function [7, 8]. Using diffusion kurtosis imaging (DKI) [58] and fixel-based analysis (FBA) [59], reduced axonal density, marked by decreased axonal water fraction, has also been suggested within the CC and long-range association tracts in adults with autism [4, 49]. The exact cause of autism remains unclear; however, some genetic factors might also contribute to axon alterations in autism as recently reviewed [60]. Indeed, mutations in the chromodomain helicase DNA binding protein 8 gene, one of the most commonly reported mutations in autism, have been associated with reduced axon and dendritic growth in humans, resulting in neuronal deficits that can contribute to autism pathophysiology [61]. Loss of axon integrity may result in reduced information processing speed in autistic participants [7]. This is reinforced by the fact that NDI was moderately inversely correlated (although the correlation was not established after correction for multiple comparisons) with AQ-communication score in the left ATR, SLF, and UF, the language and social processing-related tracts that have an impact on communication, whose deficits are the core of autism [51, 62, 63]. Furthermore, left hemisphere regions are critical for language functions, especially in right-handed individuals (all participants studied were right-handed) [64]. Taken together, we can assume that axonal loss is a likely pathological substrate for autistic symptoms, particularly in communication impairment.

The higher ISOVF observed in individuals with autism indicated increased extracellular water volume, which is expected in neuroinflammatory states [65]. Postmortem studies have demonstrated the presence of brain neuroinflammation in patients with autism, as shown by marked activation of astrocytes and microglia together with abnormal chemokine and cytokine levels, such as IL-6, IL-8, IFN-γ, TNF-α, and TGF-β1 [9, 66–68]. Neuroinflammation is expected to affect the interstitial extraneuronal space where the microglia and other immunoreactive cells mediate neuroinflammation [69], thereby increasing the isotropic diffusion of extracellular water content [65]. However, histologically confirming ISOVF as a neuroinflammatory marker is not feasible since it is an active physiological process not observed in fixed samples [70]. A recent longitudinal study in transgenic rats with Alzheimer’s disease (TgF344-AD) found that the evolution of ISOVF changes corresponds to the inflammatory burden [71]. Furthermore, in humans, a positive correlation was observed between a diffusion MRI marker of extracellular free-water and positron emission tomography imaging of the translocator protein, a putative neuroinflammatory marker [72]. Some previous studies have also demonstrated higher ISOVF in the brain of patients with multiple sclerosis [73, 74], Parkinson’s disease [75], and hypertension [76], where neuroinflammation is known to play a crucial role in the disease process.

Interestingly, NDI and ISOVF changes in individuals with autism were observed in distinct WM areas. Lower NDI was mainly demonstrated in the right hemisphere and anterior parts of the CC and left hemisphere. Conversely, higher ISOVF was shown in the posterior parts of the CC and left hemisphere. In line with our findings, asymmetry of WM diffusion abnormalities with greater differences in specific parts of the brain has also been observed in adults with autism. However, previous DTI, DKI, and FBA studies yielded mixed results, such as left-anterior [2], right-anterior [5], or right-posterior [49] hemisphere dominance or bilateral findings [4]. Inconsistency among previous findings is probably due to various factors including the heterogeneity of samples, such as age, sex, and handedness (including only either right- or left-handed or both right- and left-handed participants), and technical limitations. The loss of normal interhemispheric asymmetry is one of the most replicated findings in autism [12, 77, 78], indicating that the underlying pathological process is rather asymmetrical in individuals with autism. Our findings with NODDI suggest the possible relation between the asymmetricity in autism and pathological conditions of different levels. Evidence shows that the typical rightward cerebral asymmetry is associated with social reciprocity in autism [79]. Furthermore, microglia activation is the first sign of neuroinflammation; when activated, microglia can cause neuronal dysfunction and cell death [9]. To summarize, we can speculate that an increase in extracellular free-water and a decrease in neurite density in autism occurs via separate trajectories, and their detection might depend on scan timing. For instance, changes in ISOVF might precede those in NDI. Similar observations have been demonstrated in studies on patients with Parkinson’s disease [80] and hypertension [76]. Therefore, future longitudinal studies that fully depict the trajectory of NODDI changes in the brain of individuals with autism are warranted.

DTI evidence on higher FA with higher MD and RD in individuals with autism is consistent with those of previous studies [12, 13]. Widespread increased MD and RD for nearly all tracts showed NDI and ISOVF changes, indicating that both metrics are influenced by neurite loss and increased extracellular free-water. In contrast, increased FA was observed to a much lesser extent than NDI and ISOVF, showing the inconsistency of DTI results. As mentioned in the Introduction section, DTI is reportedly sensitive but not specific to microstructural changes [14, 81]. Furthermore, other methodological challenges were associated with DTI. First, the DTI model did resolve multiple fiber orientations in regions of crossing/kissing fibers [82]. Second, RD may provide an acceptable approximation if the voxel includes a healthy fiber bundle. If the signal-to-noise ratio is low, if crossing fibers are present, or if pathology causes decreased anisotropy, such an approach can result in misinterpretation of results [83]. Indeed, our LDA-LOOCV results showed that the NDI and ISOVF measured by NODDI had higher diagnostic accuracy, sensitivity, and specificity than DTI metrics (i.e., FA, MD, and RD, enhancing their use as robust imaging biomarkers in autism.

In the exploratory analysis, no significant difference was demonstrated in cortical structural measurements between autism and TD groups. Our results were consistent with those of previous studies reporting the dynamic pattern of abnormalities in the cortical thickness of children and adults with autism. A widespread cortical thickness increase was demonstrated in children with autism compared with TD, whereas adults with autism showed an increased rate of cortical thinning, resulting in the absence of differences with TD [26, 84]. This could be a potential indicator that WM microstructural alterations are more prevalent in adults with autism.

## Limitations

Some limitations exist in the current study. First, an absence of histopathological confirmation limited the interpretation of our findings. Second, this study has a relatively small sample size, which might have limited the power of statistical analyses and resulted in false positive or negative findings. We suspect that a relatively small sample size has reduced the statistical power, which prevented the association between NDI and AQ-communication scores from being statistically significant after correction for multiple comparisons. Therefore, the relationship between NODDI metrics and clinical scores should be carefully interpreted. Third, the age range of participants is wide (from 19 to 53 years), which increases the heterogeneity of participants. Indeed, the pattern of WM abnormality varies across the age range of individuals with autism [3, 38]. Since age was included as a nuisance covariate and no significant difference in age was observed between autism and TD groups, the impact of the wide age range was minimized. Besides age, both sexes were also included. As sex has been assumed to have an impact on results [85], it was included as a nuisance covariate. To assess the influence of sex in our results, supplementary TBSS analysis was performed between male and female individuals with autism, and no significant difference was observed. However, owing to the small sample size of female participants, the results should be cautiously interpreted.

## Conclusion

Our results suggest that NODDI metrics might be useful as imaging biomarkers for diagnosing autism in adults with an accuracy higher than that of DTI. Furthermore, NODDI allows the interpretation of previous findings on diffusion tensor metrics changes in the WM of individuals with autism. NDI and ISOVF changes might reflect neuronal loss and neuroinflammation, respectively, within the commissural and long-range association tracts in adults with autism. Our findings might also suggest that the neuronal loss within the language and social processing-related tracts is the underlying pathology of communication impairment in autism. Future histological studies should investigate the correlation between NODDI measures and WM pathological changes in autism. Therefore, an improved knowledge of the pathogeneses of autism may result in an optimized therapeutic strategy.

## Data Availability

Data used in the current study are available from the corresponding author on reasonable request.

## Abbreviations

AD: Axial diffusivity
ALIC: Anterior limb of internal capsule
ATR: Anterior thalamic radiation
AQ: Autism-spectrum quotient
CC: Corpus callosum
CCG: Cingulum cingulate gyrus
CSF: Cerebrospinal fluid
CST: Corticospinal tract
DKI: Diffusion kurtosis imaging
DTI: Diffusion tensor imaging
EQ: Empathizing quotient
FA: Fractional anisotropy
FBA: Fixel-based analysis
FOV: Field of view
FSL: FMRIB software library
FEW: Family-wise error
GLM: General linear model
GM: Gray matter
ICV: Intracranial volume
IFOF: Inferior fronto-occipital fasciculus
ILF: Inferior longitudinal fasciculus
ISOVF: Isotropic volume fraction
LDA: Linear discriminant analysis
LOOCV: Leave-one-out cross-validation
MD: Mean diffusivity
NDI: Neurite density index
NODDI: Neurite orientation dispersion and density imaging
ODI: Orientation dispersion index
PLIC: Posterior limb of internal capsule
RD: Radial diffusivity
ROI: Region-of-interest
SLF: Superior longitudinal fasciculus
SQ: Systemizing quotient
TBSS: Tract-based spatial statistics
TD: Typically developing subjects
TE: Echo time
TFE: Turbo field echo
TR: Repetition time
UF: Uncinate fasciculus
WM: White matter
